# Profound immune suppression and exhaustion characterize refractory mycoplasma pneumoniae pneumonia in children

**DOI:** 10.3389/fimmu.2026.1839837

**Published:** 2026-06-09

**Authors:** Xiaolin Ma, Yuting Wu, Feng He, Hailan Yao, Ling Cao, Chunmei Zhu

**Affiliations:** 1 Department of Respiratory Medicine, Capital Center for Children’s Health, Capital Medical University, Capital Institute of Pediatrics, Beijing, China; 2 Department of Biochemistry and Immunology, Capital Center for Children’s Health, Capital Medical University, Capital Institute of Pediatrics, Beijing, China

**Keywords:** adaptive immunity, children, immune suppression, lymphocyte subsets, plasmablasts, predictive model, refractory *Mycoplasma pneumoniae* pneumonia

## Abstract

**Background:**

The mechanisms underlying refractory *Mycoplasma pneumoniae* (*M. pneumoniae*) pneumonia (RMPP) and its association with adaptive immune dysfunction remain incompletely defined. This study investigated clinical features and peripheral lymphocyte profiles in children with RMPP versus those with common *M. pneumoniae* pneumonia (CMPP) to identify immune disturbances that may serve as early predictive indicators.

**Methods:**

Of 622 children diagnosed with *M. pneumoniae* pneumonia between January 2019 and January 2024, 139 with single infection were enrolled: 72 RMPP and 67 CMPP. Clinical data and laboratory inflammatory markers were collected. Peripheral lymphocyte subsets (percentages and absolute counts) were determined by flow cytometry. Independent risk factors for RMPP were identified using multivariate logistic regression. A combined diagnostic model was constructed and evaluated by area under the receiver operating characteristic curve.

**Results:**

Children with RMPP exhibited more severe clinical manifestations than those with CMPP, including prolonged high fever, higher rates of severe disease and glucocorticoid use, and extensive lung involvement (multilobar infiltration, pleural effusion). Laboratory tests revealed an accentuated systemic inflammatory response (elevated C-reactive protein, procalcitonin) in RMPP. Immunologically, RMPP was characterized by extensive reductions in absolute lymphocyte counts—encompassing T cells (particularly helper and naïve subsets), B cells, regulatory T cells, and γδ T cells—despite minimal percentage differences in most subsets. Percentage-wise, RMPP showed decreased helper T cells but increased proportions of helper effector memory T cells, terminally differentiated effector memory helper/cytotoxic T cells re-expressing CD45RA. Multivariate analysis identified older age, longer fever duration, pleural effusion, and decreased absolute plasmablast count as independent predictors of RMPP. A four-indicator combined model demonstrated good discrimination (area under the curve, 0.81; 95% confidence interval, 0.74–0.88); an optimal threshold of 0.48 yielded 96% sensitivity and 52% specificity.

**Conclusion:**

RMPP involves profound adaptive immunosuppression, marked by widespread reduction in total lymphocytes and key functional subsets—particularly plasmablasts—alongside an exhausted/memory phenotypic shift in specific T cells. Plasmablast reduction represents a novel immunological marker for predicting RMPP. A model integrating plasmablast count, age, fever duration, and pleural effusion holds promise for early RMPP identification, providing valuable insights into its immunopathogenesis and informing early warning strategies.

## 1 Introduction

*Mycoplasma pneumoniae* (*M. pneumoniae*) is a leading cause of community-acquired pneumonia in children. The rising incidence of refractory *M. pneumoniae* pneumonia (RMPP) has garnered significant clinical attention. Characterized by a poor response to macrolide antibiotics, prolonged course, and progressive clinical and radiographic deterioration, RMPP is frequently complicated by severe conditions such as necrotizing pneumonia, pulmonary embolism, and acute asthma exacerbations, thereby increasing the disease burden on pediatric patients and their families (1). While the precise mechanisms remain elusive, current evidence implicates not only pathogen-related factors (e.g., drug resistance, virulence) but also aberrant host immune responses—particularly excessive inflammation and immune dysregulation—in RMPP pathogenesis (1–3). This maladaptive immune activation may exacerbate tissue damage and complicate the clinical course. However, systematic analyses of specific immune cell subset alterations, especially absolute lymphocyte counts and their correlation with disease severity and prognosis in RMPP, are still lacking and warrant further investigation (4).

A comprehensive comparison of immune status, particularly the distribution of lymphocyte subsets, between children with RMPP and those with common *M. pneumoniae* pneumonia (CMPP) is crucial for elucidating RMPP immunopathogenesis. Such an analysis could provide key laboratory evidence and identify potential early warning indicators for high-risk patients, guiding individualized interventions like immunomodulatory therapy. Therefore, this retrospective case-control study was designed to conduct an in-depth analysis of absolute counts of peripheral blood lymphocyte subsets—including T cells (e.g., total, helper, cytotoxic and their naïve/memory subsets, double-positive T cells (DPT), double-negative T cells (DNT), regulatory T cells (Treg), γδ T cells and B cells (e.g., total, naïve, transitional B cells, and plasmablasts). Our objective was to delineate the unique immune dysregulation landscape in RMPP and, based on these findings, to identify independent risk factors for its development. Furthermore, we aimed to construct a diagnostic prediction model that integrates key clinical and immunological indicators. This work is expected to offer novel insights and a foundation for the early identification and precise management of RMPP.

## 2 Materials and methods

### 2.1 Study subjects and data collection

We screened the inpatient database of the Respiratory Department at Capital Center for Children’s Health, Capital Medical University, for children admitted between January 2019 and January 2024. A total of 622 children met the diagnostic criteria for *M. pneumoniae* pneumonia (MPP), as confirmed by a positive nucleic acid test for *M. pneumoniae*. Children were classified as having RMPP if, after more than seven days of standard macrolide therapy, they presented with persistent fever, worsening clinical signs and radiographic findings, and/or extrapulmonary complications. The remaining children were classified as having CMPP. Exclusion criteria were congenital immunodeficiency, malignancy or hematologic diseases, and co-infection with other pathogens (bacteria or viruses). Ultimately, 139 children with a single *M. pneumoniae* infection were enrolled in the final analysis (RMPP group: n = 72; CMPP group: n = 67). Demographic data, underlying diseases, symptoms, signs, and laboratory parameters were collected for all participants. Chest CT images were reviewed to document features including multilobar infiltration, pleural effusion, and atelectasis. This retrospective study was approved by the Ethics Committee of Capital Institute of Pediatrics (Approval No. SHERLL2023076). The requirement for informed consent was waived due to the use of anonymized retrospective data.

### 2.2 Research methods

#### 2.2.1 Immunophenotyping of peripheral blood lymphocytes

Blood samples were collected at the time of hospital admission, but after at least 7 days of macrolide therapy. A 2 mL sample of ethylenediaminetetraacetic acid-anticoagulated peripheral blood was collected from each enrolled child. Samples were processed within two hours of collection using a dual-platform approach. Absolute lymphocyte counts were obtained using a hematology analyzer (Sysmex XN-9000). Lymphocyte subset proportions were determined by flow cytometry (BD FACSCanto II™) (5). Lymphocyte subset reference ranges were age-standardized using established pediatric normative data (6–8). Absolute counts and percentages of lymphocyte subsets were interpreted according to age-specific reference intervals. Briefly, 50 μL of whole blood was incubated with a pre-mixed antibody panel for 20 minutes in the dark. Red blood cells were lysed using BD FACS™ Lysing Solution. Cells were then washed, fixed, and acquired. Isotype controls were used for gating. Relative percentages were analyzed using FACSDiva™ software, and absolute counts (cells/μL) were calculated by multiplying these percentages by the total lymphocyte count. The immunophenotyping analysis encompassed a panel of 21 lymphocyte subsets: T cells (CD45+CD3+), helper T cells (CD4; CD3+CD4+), naïve helper T cells (CD4 naïve; CD3+CD4+CD45RA+CD27+), central memory helper T cells (CD4 CM; CD3+CD4+CD45RA-CD27+), effector memory helper T cells (CD4 EM; CD3+CD4+CD45RA-CD27-), terminally differentiated effector memory helper T cells re-expressing CD45RA (CD4 TEMRA; CD3+CD4+CD45RA+CD27-), cytotoxic T cells (CD8; CD3+CD8+), naïve cytotoxic T cells (CD8 naïve; CD3+CD8+CD45RA+CD27+), central memory cytotoxic T cells (CD8 CM; CD3+CD8+CD45RA-CD27+), effector memory cytotoxic T cells (CD8 EM; CD3+CD8+CD45RA-CD27-), terminally differentiated effector memory cytotoxic T cells re-expressing CD45RA (CD8 TEMRA; CD3+CD8+CD45RA+CD27-), DNT (CD3+TCRαβ+CD4-CD8-), DPT (CD3+CD4+CD8+), γδ T cells (CD3+TCRγδ+), Treg (CD4+CD25+CD127^low^), B cells (CD19+), naïve B cells (CD19+CD27-IgD+), memory B cells (CD19+CD27+IgD-), transitional B cells (CD19+CD24++CD38++), plasmablasts (CD19+CD24-CD38++), and natural killer cells (CD3-CD16+CD56+).

#### 2.2.2 Detection of respiratory pathogen

Sputum or bronchoalveolar lavage fluid samples were collected from each child. Nucleic acids were extracted from 140 µL of sample using the QIAamp^®^ Viral RNA Mini Kit (QIAGEN, Hilden, Germany) (5). The eluate (60 µL) was stored at −80 °C. Viral pathogens were detected using the NxTAG™ Respiratory Pathogen Panel assay (Luminex Molecular Diagnostics Inc., Toronto, Canada), which includes influenza A/B, respiratory syncytial virus, human adenovirus, metapneumovirus, rhinovirus, bocavirus, parainfluenza virus, and coronavirus. Bacterial pathogens were identified using a Pathogen Nucleic Acid Detection Kit (CapitalBio Technology, Beijing, China), which covers *Streptococcus pneumoniae*, *Haemophilus influenzae*, *Staphylococcus aureus*, methicillin-resistant *Staphylococcus*, *Escherichia coli*, *Klebsiella pneumoniae*, *Pseudomonas aeruginosa*, *Acinetobacter baumannii*, *Stenotrophomonas maltophilia*, *Mycobacterium tuberculosis* complex, and *Legionella pneumophila*. *M. pneumoniae* and *Chlamydia pneumoniae* were detected using both the NxTAG™ Respiratory Pathogen Panel assay and the CapitalBio Pathogen Nucleic Acid Detection Kit. Conventional bacterial culture was also performed on all respiratory samples.

### 2.3 Statistical analysis

Continuous variables following a normal distribution are expressed as mean ± standard deviation and were compared using the independent samples t-test. Non-normally distributed continuous variables are presented as median (interquartile range) and were compared using the Mann–Whitney U test. Categorical variables are presented as counts (percentages) and were compared using the χ² test or Fisher’s exact test, where appropriate. Multivariate logistic regression analysis was performed to identify independent risk factors for RMPP, with odds ratios (OR) and their 95% confidence intervals (CI) calculated. The diagnostic performance of potential predictors was evaluated using receiver operating characteristic curve analysis, from which the area under the curve, sensitivity, and specificity were derived. All statistical analyses were conducted using R software (version 4.3.3) running in RStudio Desktop (2023.12.1 Build 402, Ocean Storm Release). A two-sided p-value < 0.05 was considered statistically significant.

## 3 Results

### 3.1 Comparison of clinical characteristics between RMPP and CMPP

Regarding baseline features (**Table 1**), children in the RMPP group were significantly older than those in the CMPP group (mean age: 97.04 ± 33.19 months vs. 75.90 ± 34.30 months, *p* < 0.001), whereas sex distribution was comparable between the groups. The RMPP group exhibited more severe clinical manifestations. The proportion of patients classified as having severe disease was significantly higher in the RMPP group (98.6% vs. 89.6%, *p* = 0.029), and a greater proportion experienced progressive clinical worsening (59.72% vs. 26.87%, *p* < 0.001). The duration of fever was significantly longer in the RMPP group (9.42 ± 4.32 days vs. 6.31 ± 3.72 days, *p* < 0.001), as was the number of days with high fever (≥39°C) [5.00 (2.25, 8.00) days vs. 2.00 (0.00, 6.00) days, *p* = 0.001]. Furthermore, glucocorticoid use was significantly more frequent in the RMPP group (69.44% vs. 43.28%, *p* = 0.002). No significant between-group differences were observed in the prevalence of underlying diseases, dyspnea, or atelectasis.

**Table 1:** Comparison of clinical manifestations and laboratory indicators between children with refractory *Mycoplasma pneumoniae* pneumonia (RMPP) and those with common *Mycoplasma pneumoniae* pneumonia (CMPP).

Clinical indicator	RMPP (n=72)	CMPP (n=67)	*P*-value
Male	37 (51.39%)	30 (44.78%)	0.44
Age (months)	97.04 ± 33.19	75.90 ± 34.30	<0.001
Underlying disease	3 (4.17%)	5 (7.46%)	0.40
Glucocorticoids	50 (69.44%)	29 (43.28%)	0.002
Severe cases	71 (98.61%)	60 (89.55%)	0.029
Length of hospital stay (days)	6.13 ± 3.66	5.04 ± 2.76	0.053
Duration of fever (days)	9.42 ± 4.32	6.31 ± 3.72	<0.001
High fever ≥39 °C (days)	5.00 (2.25, 8.00)	2.00 (0.00, 6.00)	0.001
Dyspnea	7 (9.72%)	11 (16.42%)	0.24
Hypoxemia	13 (18.06%)	6 (8.86%)	0.12
Respiratory failure	1 (1.39%)	1 (1.49%)	1.00
Progressive clinical worsening	43 (59.72%)	18 (26.87%)	<0.001
Plastic bronchitis	0 (0.00%)	3 (4.48%)	0.11
Multilobar infiltration	59 (81.95%)	43 (64.18%)	0.018
Pleural effusion	15 (20.83%)	3 (4.48%)	0.004
Atelectasis	5 (6.94%)	7 (10.45%)	0.46
Bronchoscopy	46 (63.89%)	42 (62.69%)	0.88
Mechanical ventilation	1 (1.39%)	0 (0.00%)	1.00
White blood cell count (×10^9^/L)	8.26 (6.13, 12.50)	8.28 (6.56, 11.3)	0.88
Hemoglobin (g/L)	129.58 ± 11.75	126.69 ± 10.90	0.14
Platelet count (×10^9^/L)	324.00 (234.25, 420.75)	354.00 (273.00, 413.00)	0.29
Neutrophil count (×10^9^/L)	5.22 (3.22, 7.88)	4.58 (2.81, 6.77)	0.065
Neutrophil-to-lymphocyte ratio	2.55 (1.48, 4.47)	1.48 (0.80, 2.53)	<0.001
C-reactive protein (mg/L)	23.60 (15.87, 46.18)	13.10 (6.00, 26.31)	<0.001
Procalcitonin (ng/mL)	0.09 (0.05, 0.14)	0.06 (0.05, 0.11)	0.019
Alanine aminotransferase (U/L)	14.75 (11.18, 25,65)	11.80 (9.50, 36.92)	0.004
Aspartate aminotransferase (U/L)	24.55 (19.20, 30.80)	23.70 (19.30, 30.10)	0.93
Lactate dehydrogenase (U/L)	294.00 (231.00, 366.00)	276.00 (237.00, 336.00)	0.44
α-Hydroxybutyrate dehydrogenase (U/L)	216.00 (165.25, 269.75)	198.00 (176.00, 242.00)	0.65
D-dimer (mg/L FEU)	0.76 (0.40, 1.71)	0.55 (0.35, 1.83)	0.057

Laboratory markers of inflammation were generally more elevated in the RMPP group. The lymphocyte count was significantly lower in the RMPP group [2.17 (1.66, 2.72) ×10^9^/L vs. 2.87 (2.24, 4.32) ×10^9^/L, *p* < 0.001], while the neutrophil-to-lymphocyte ratio was significantly higher [2.55 (1.48, 4.47) vs. 1.48 (0.80, 2.53), *p* < 0.001]. No significant differences were observed in white blood cell count or hemoglobin levels. Levels of both C-reactive protein [23.60 (15.87, 46.18) mg/L vs. 13.10 (6.00, 26.31) mg/L, *p* < 0.001] and procalcitonin [0.09 (0.05, 0.14) ng/mL vs. 0.06 (0.05, 0.11) ng/mL, *p* = 0.019] were significantly elevated in the RMPP group. Alanine aminotransferase levels were also higher in the RMPP group [14.75 (11.18, 25.65) U/L vs. 11.80 (9.50, 36.92) U/L, *p* = 0.004], whereas no significant differences were found for lactate dehydrogenase, D-dimer, or α-hydroxybutyrate dehydrogenase. Significant differences were also observed in imaging features. The incidence of multilobar infiltration was higher in the RMPP group (81.95% vs. 64.18%, *p* = 0.018), as the incidence of pleural effusion (20.83% vs. 4.48%, *p* = 0.004).

### 3.2 Analysis of lymphocyte subset immunological features in RMPP and CMPP

#### 3.2.1 Lymphocyte subset percentages

**Figure 1:**
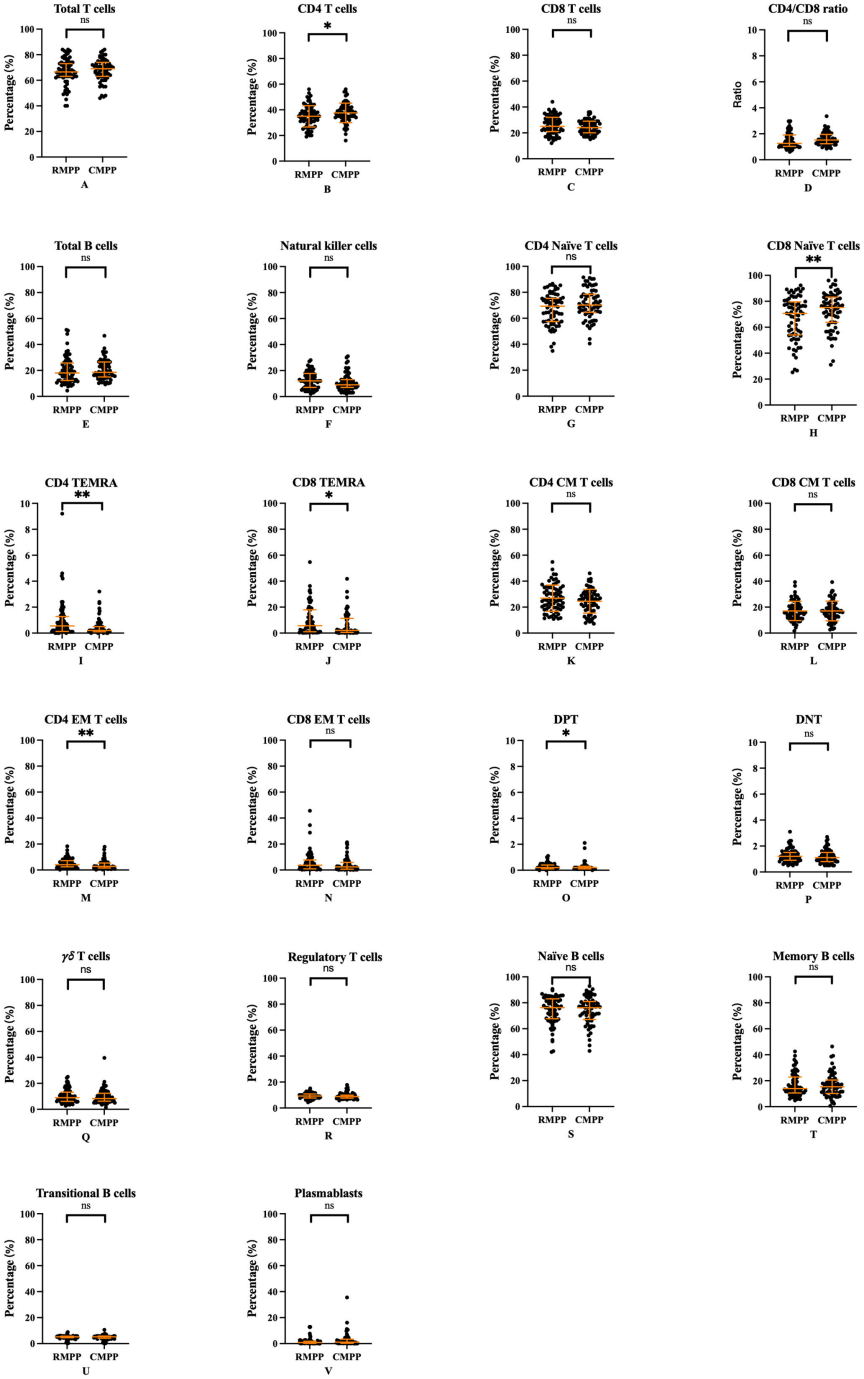
Comparison of percentages of lymphocyte subsets between children with refractory *Mycoplasma pneumoniae* pneumonia (RMPP) and common *Mycoplasma pneumoniae* pneumonia (CMPP). **(A)** Total T cells, **(B)** CD4 T cells, **(C)** CD8 T cells, **(D)** CD4/CD8 ratio, **(E)** Total B cells, **(F)** Natural killer cells, **(G)** CD4 naïve T cells, **(H)** CD8 naïve T cells, **(I)** CD4 TEMRA, **(J)** CD8 TEMRA, **(K)** CD4 CM T cells, **(L)** CD8 CM T cells, **(M)** CD4 EM T cells, **(N)** CD8 EM T cells, **(O)** DPT, **(P)** DNT, **(Q)** γδ T cells, **(R)** Regulatory T cells, **(S)** Naïve B cells, **(T)** Memory B cells, **(U)** Transitional B cells, **(V)** Plasmablasts. Data are presented as mean ± standard deviation for normally distributed variables or as median with interquartile range (IQR) for non-normally distributed variables. Statistical comparisons were performed using the independent samples t-test or Mann–Whitney U test, as appropriate. * and ** indicate p< 0.05 and p < 0.01, respectively. CM, central memory; DNT, double-negative T cells; DPT, double-positive T cells; EM, effector memory; ns, no significance; TEMRA, terminally differentiated effector memory T cells re-expressing CD45RA.

No significant differences were observed between the RMPP and CMPP groups for most lymphocyte subtypes (**Figure 1**). However, among helper T lymphocyte subsets, the percentage of CD4 cells was significantly lower in the RMPP group. Conversely, the percentages of CD4 TEMRA and CD4 EM T cells were significantly higher in the RMPP group. Among cytotoxic T lymphocyte subsets, the percentage of CD8 TEMRA was also significantly elevated in the RMPP group. Additionally, the percentage of DPT was higher in the RMPP group. No significant differences were observed for other major subsets, including total T cells, CD4 naïve, CD8 T cells, γδ T cells, Treg, B cells and their subsets, or natural killer cells. **Supplementary Table 1** provides detailed subset comparisons and statistical values.

#### 3.2.2 Absolute lymphocyte counts

**Figure 2:**
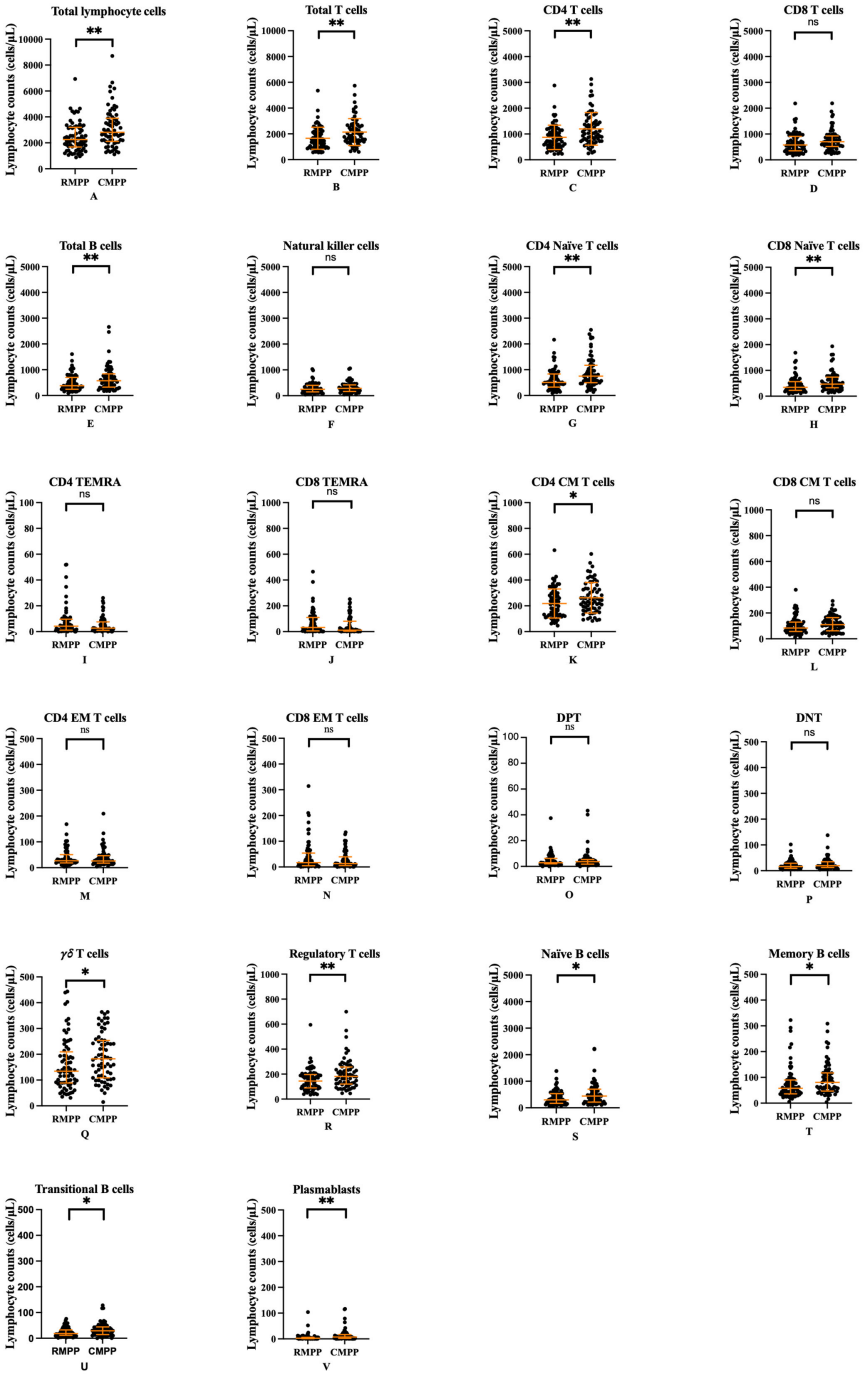
Comparison of absolute counts of lymphocyte subsets between children with refractory *Mycoplasma pneumoniae* pneumonia (RMPP) and common *Mycoplasma pneumoniae* pneumonia (CMPP). **(A)** Total lymphocyte, **(B)** Total T cells, **(C)** CD4 T cells, **(D) **CD8 T cells, **(E)** Total B cells, **(F)** Natural killer cells, **(G)** CD4 naïve T cells, **(H)** CD8 naïve T cells, **(I)** CD4 TEMRA, **(J)** CD8 TEMRA, **(K)** CD4 CM T cells, **(L)** CD8 CM T cells, **(M)** CD4 EM T cells, **(N)** CD8 EM T cells, **(O)** DPT, **(P)** DNT, **(Q)** γδ T cells (CD3^+^TCRγδ^+^), **(R)** Regulatory T cells, **(S)** Naïve B cells, **(T)** Memory B cells, **(U)** Transitional B cells, **(V)** Plasmablasts. Data are presented as mean ± standard deviation for normally distributed variables or as median with interquartile range (IQR) for non-normally distributed variables. Statistical comparisons were performed using the independent samples t-test or Mann–Whitney U test, as appropriate. * and ** indicate p< 0.05 and p < 0.01, respectively. CM, central memory; DNT, double-negative T cells; DPT, double-positive T cells; EM, effector memory; ns, no significance; TEMRA, terminally differentiated effector memory T cells re-expressing CD45RA.

In contrast to the percentage data, the RMPP group exhibited widespread numerical reductions in absolute lymphocyte counts (**Figure 2**). The total lymphocyte count was significantly lower in the RMPP group, as was the absolute total T lymphocyte count. Further analysis of T cell subsets showed significantly lower absolute counts in the RMPP group for: CD4, CD4 naïve, CD4 CM, and CD8 naïve. Additionally, absolute counts of Treg and γδ T cells were significantly reduced in the RMPP group. The absolute B cell count was significantly lower in the RMPP group. Among B cell subsets, absolute counts were significantly reduced for memory B cells, naïve B cells, transitional B cells, and plasmablasts. In contrast, the absolute natural killer cell count showed no significant difference between the groups. Detailed subset comparisons and statistical values are provided in **Supplementary Table 2**.

### 3.3 Independent risk factors and diagnostic model for RMPP

Multivariate logistic regression analysis was performed to identify independent predictors of RMPP. As shown in **Table 2**, independent predictive factors for RMPP were: older age (OR = 1.02, 95% CI: 1.004–1.03, *p* = 0.011), longer duration of fever (OR = 1.24, 95% CI: 1.09–1.40, *p* = 0.001), presence of pleural effusion (OR = 9.86, 95% CI: 1.59–61.32, *p* = 0.014), and lower plasmablast count (OR = 0.97, 95% CI: 0.95–0.997, *p* = 0.031). Sex and neutrophil-to-lymphocyte ratio were not retained as independent predictors in the final model.

**Table 2:** Multivariate logistic regression analysis of refractory *Mycoplasma pneumoniae* pneumonia (RMPP) in children.

Variables	Regression coefficient	Odds ratio	95% confidence interval		*P*-value
			Lower limit	Upper limit	
Male	-0.33	0.72	0.31	1.66	0.44
Age (months)	0.017	1.02	1.004	1.03	0.011
Duration of fever (days)	0.21	1.24	1.09	1.40	0.001
Progressive clinical worsening	0.80	2.22	0.91	5.38	0.079
Pleural effusion	2.29	9.86	1.59	61.32	0.014
Plasmablasts (cells/üL)	-0.029	0.97	0.95	0.997	0.031
Neutrophil-to-lymphocyte ratio	0.21	1.24	0.96	1.60	0.10
Constant	-3.70				

**Figure 3:**
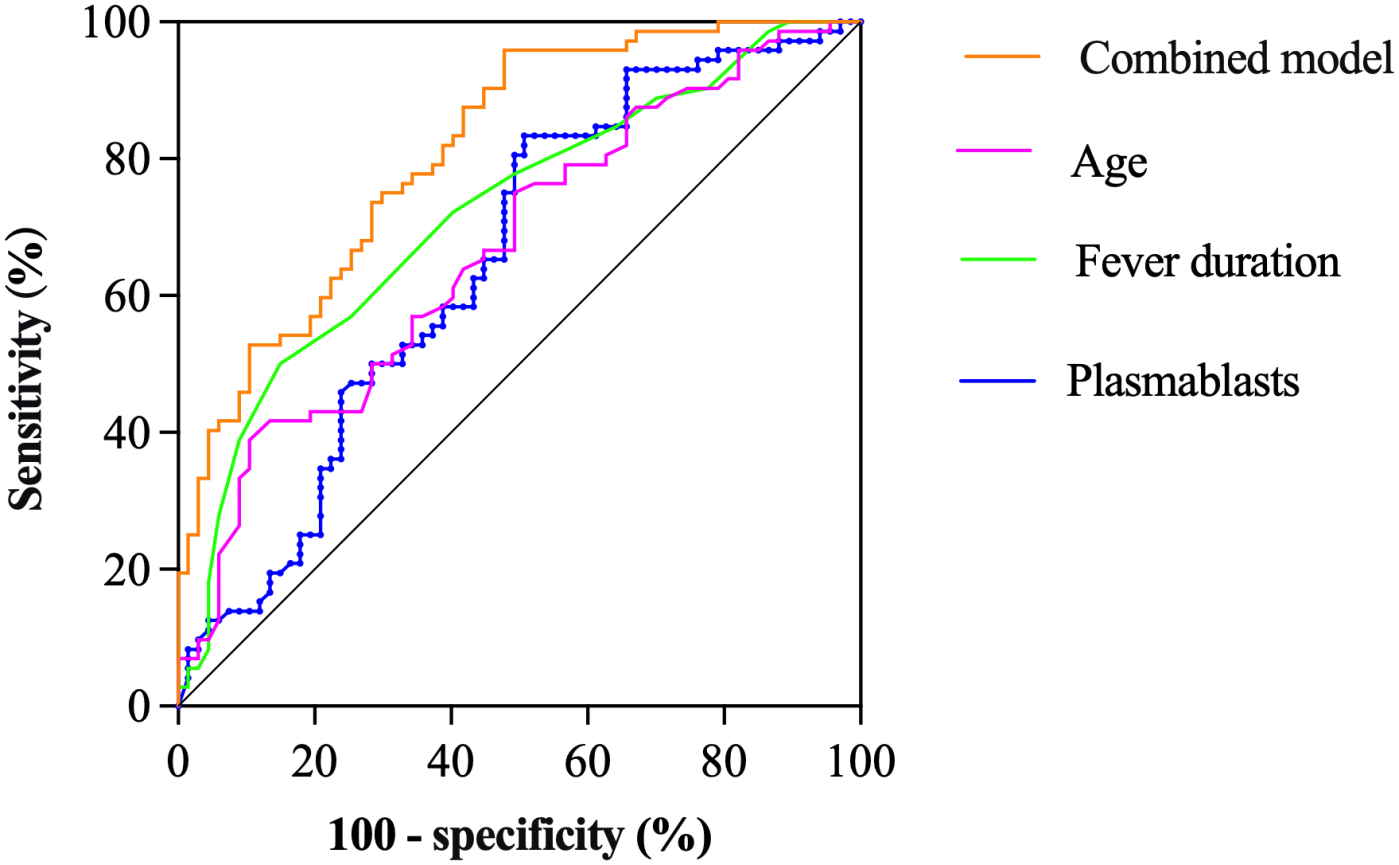
Receiver operating characteristic curves for individual predictors and the combined diagnostic model for refractory *Mycoplasma pneumoniae* pneumonia (RMPP). The combined model incorporated four independent predictors: age, fever duration, pleural effusion, and absolute plasmablast count. The area under the curve for the combined model was 0.81 (95% CI: 0.74–0.88), indicating good discriminatory ability. The area under the curve for individual predictors were: plasmablast count, 0.65 (95% CI: 0.56–0.74); age, 0.67 (95% CI: 0.58–0.76); and fever duration, 0.72 (95% CI: 0.64–0.81). CI, confidence interval.

A receiver operating characteristic curve was plotted to evaluate the predictive performance of a combined diagnostic model incorporating these four independent factors (**Figure 3**). The area under the curve for each individual factor was: plasmablast count, 0.65 (95% CI: 0.56–0.74, *p* = 0.0023); age, 0.67 (95% CI: 0.58–0.76, *p* = 0.0007); and fever duration, 0.72 (95% CI: 0.64–0.81, *p* < 0.0001). The model combining all four predictive factors achieved an area under the curve of 0.81 (95% CI: 0.74–0.88, *p* < 0.0001), indicating good discriminatory ability Based on the receiver operating characteristic curve analysis, the Youden index was maximized at a probability threshold of 0.48 for the combined model. At this threshold, the model yielded a sensitivity of 96% and a specificity was 52%.

## 4 Discussion

**Figure 4:**
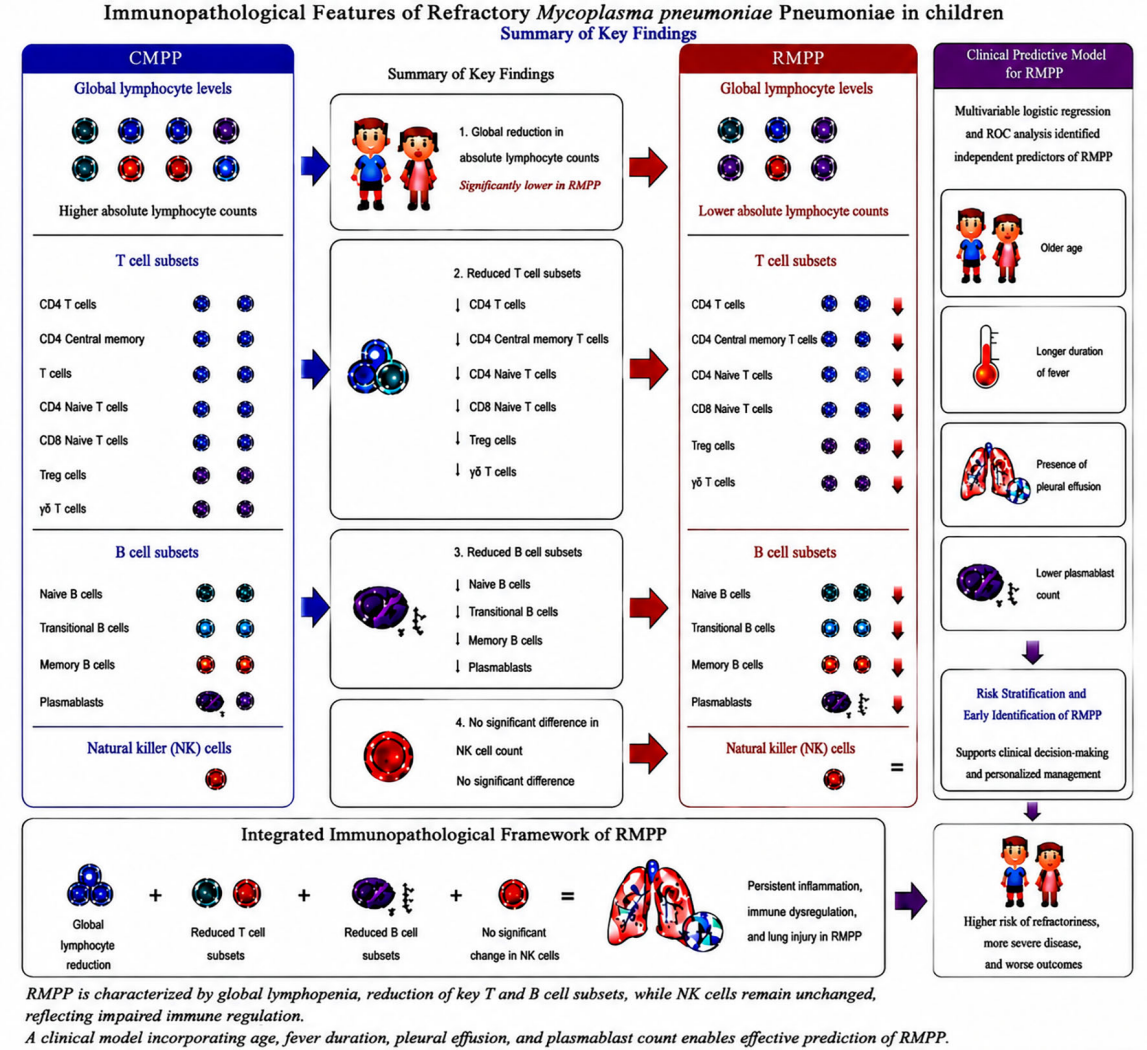
Schematic overview of immune alterations and the clinical predictive model for refractory *Mycoplasma pneumoniae* pneumonia (RMPP) in children. CMPP, common *Mycoplasma pneumoniae* pneumonia.

By comprehensively immunophenotyping peripheral blood lymphocytes from children with RMPP and CMPP, this study delineated a distinct immune landscape associated with RMPP and explored its potential relationship with disease severity. We demonstrate that children with RMPP exhibit profound adaptive immunosuppression and dysfunction, characterized by a global numerical reduction in T and B cells, an exhausted phenotype in specific T cell subsets, and a concomitant decrease in immunoregulatory populations (**Figure 4**). Collectively, these findings offer new insights into the immunopathogenesis of RMPP.

### 4.1 Reconciling discrepancies between absolute counts and percentages

Our findings reveal a critical distinction between relative proportions (percentages) and absolute numbers of lymphocyte subsets in children with RMPP—a distinction essential for accurately interpreting the nature of the immune dysregulation.

The percentage data indicate a compositional shift within the T cell compartment. The decreased proportion of CD4 T cells, coupled with increased proportions of CD4 TEMRA, CD4 CM, and CD8 TEMRA, suggests a phenotypic skewing toward memory and exhausted subsets. This implies that, relative to the overall T cell pool, the remaining T cells in children with RMPP are more likely to exhibit a differentiated, potentially exhausted phenotype. However, the absolute count data provide a more comprehensive picture by revealing the overall quantitative loss. The significantly lower total lymphocyte and absolute T cell counts demonstrate true depletion of these cells from the peripheral blood. Critically, the absolute counts of key subsets—including total CD4, CD4 naïve, CD4 CM, and CD8 naïve —are also significantly reduced.

The apparent paradox—where the *percentage* of a subset (e.g., CD4 CM) is increased yet its *absolute count* is decreased—is explained by the profound reduction in total lymphocyte and total T cell populations. A subset’s percentage can increase not because its own numbers have risen, but simply because other subsets have declined even more dramatically, rendering it a larger fraction of a diminished total pool. In this context, the increased percentages of memory and exhausted subsets likely reflect contraction of the naïve T cell pool and relative enrichment of remaining differentiated cells, rather than true expansion. Thus, absolute count reductions serve as the more direct indicator of immune suppression, revealing a genuine loss of immune effector cells. Percentage shifts, while valuable for understanding the functional phenotype of the remaining cells, must be interpreted in light of this overall numerical depletion. This dual-layered analysis underscores that RMPP is characterized not merely by functional imbalance but by a profound, quantitative loss of adaptive immune capacity.

### 4.2 Widespread suppression of adaptive immunity: broad reduction in T and B cells

This study reveals significant T cell dysregulation in children with RMPP. Compared to the CMPP group, children with RMPP exhibited markedly reduced absolute counts of total T lymphocyte (CD3) and CD4 T cells in peripheral blood, indicating an overall impairment of T cell-mediated immunity. Given the central role of T cells in host defense against infection, such impairment likely contributes to delayed pathogen clearance and a protracted disease course (9, 10). These findings align with prior reports of impaired CD3, CD4 and CD19 counts in pediatric RMPP (11), which fails to further investigate subsets of T and B cells. Further subset analysis in present study revealed markedly reduced numbers of CD4 naïve, CD8 naïve, and CD4 CM. This reduction may compromise the capacity for early antigen recognition and the formation or maintenance of long-term immunological memory, thereby partially explaining the refractoriness of RMPP. Thus, our study provides more systematic and direct evidence—across multiple dimensions including cell numbers, subset differentiation, and functional phenotypes—for the role of T cell-mediated adaptive immunity in RMPP pathogenesis.

Children with RMPP also exhibited comprehensive defects in the B cell compartment. Total B lymphocytes, along with memory, naïve, and transitional B cell subsets as well as plasmablasts, were all significantly reduced. Plasmablasts—the effector cells derived from terminally differentiated B cells—are core executors of humoral immune responses (12). They directly neutralize pathogens via antibody secretion and participate in regulating the establishment of protective immunity; consequently, the quantity of circulating plasmablasts is a key determinant of effective pathogen clearance (13). Previous studies using single-cell RNA sequencing (scRNA-seq) have confirmed impaired function in peripheral B cell subsets, including plasmablast precursors, in mild MPP, linking such impairment to adaptive immune defects in pathogen clearing (14). Therefore, the sharp decrease in plasmablasts observed in our study strongly suggests severe suppression of the effector phase of humoral immunity. This widespread impairment across the B cell differentiation continuum likely results in insufficient production of specific antibodies, thereby weakening pathogen clearance via neutralization and opsonophagocytosis. The drastic reduction in plasmablasts is particularly critical, as it indicates severely compromised antibody production capacity. Reductions in naïve and transitional B cells may impair repertoire replenishment and early differentiation, while a paucity of memory B cells could weaken long-term humoral protection.

Collectively, these defects spanning the entire B cell differentiation process constitute the cellular basis for insufficient humoral immunity in children with RMPP, leading to a diminished capacity to clear *M. pneumoniae* through antibody-mediated mechanisms. A key contribution of our study is the first systematic depiction of universal impairment across all developmental stages of B cells in RMPP, revealing that immune dysregulation in RMPP concurrently involves both cellular (T cell) and humoral (B cell) immunity. This dual adaptive immune defect thus jointly contributes to impaired pathogen clearance and disease refractoriness.

### 4.3 T cell exhaustion: a key immunopathological mechanism

T cell exhaustion is a dysfunctional state, well-characterized in chronic infections and malignancies, defined by reduced proliferative capacity, diminished cytokine secretion, and impaired antigen responsiveness (15). Here, we report, for the first time, a significant increase in the proportion of peripheral blood exhausted T cells (encompassing both CD4 TEMRA and CD8 TEMRA) in children with RMPP, suggesting that T cell exhaustion may represent an important immunopathological mechanism in this condition. This finding aligns with a recent scRNA-seq study reporting CD8 T cell exhaustion in children with MPP (16) and provides a plausible immunological explanation for the refractoriness of RMPP. Supporting this, scRNA-seq analysis of bronchoalveolar lavage fluid from children with MPP confirmed that infection, particularly in severe cases, can induce CD8 T cell exhaustion, characterized by upregulation of exhaustion-associated transcription factors such as *PTPN6*, *PTPN11*, and *PRDM1* (16). This exhausted state is thought to potentially drive disease progression in MPP (16).

Although T cell exhaustion has been observed in MPP, its specific role in RMPP has not been systematically explored. Our study is the first to explicitly position T cell exhaustion as a central mechanistic framework for understanding RMPP. We propose that persistent *M. pneumoniae* antigen stimulation may drive antigen-specific T cells into a state of premature exhaustion, thereby impairing their ability to execute effector functions—including pathogen killing and immune coordination—and constituting a key immunological mechanism underpinning the protracted course and clinical deterioration characteristic of RMPP. Therefore, our findings not only provide novel insights into RMPP immunopathology but also suggest promising therapeutic avenues. Exploring immunomodulatory strategies aimed at reversing T cell exhaustion, such as immune checkpoint modulation, represents a compelling direction that addresses an unmet clinical need in this field.

### 4.4 Concomitant deficiency of Treg and γδ T cells: an immunoregulatory network imbalance in RMPP

In addition to adaptive immune suppression, we observed a significant reduction in peripheral blood Treg and γδ T cells in children with RMPP. Treg are essential for maintaining immune homeostasis and curbing excessive inflammation; their reduction may therefore exacerbate pulmonary immunopathology (17). Studies in models of heart failure and *S. pneumoniae* infection have demonstrated that Treg depletion or dysfunction exacerbates lung inflammation and promotes overactivation of IL-17-producing γδ T cells, illustrating how Treg deficiency can disrupt immunoregulatory balance and fuel excessive inflammation (18, 19). Furthermore, FOXP3+ natural Treg facilitate inflammation resolution and tissue repair, as evidenced in viral pneumonia models (20).

γδ T cells form a functional bridge between innate and adaptive immunity and play a critical role in early defense against pulmonary infections (21, 22). In models such as *S. pneumoniae* infection, γδ T cells interact with Treg to maintain inflammatory equilibrium; impairment of Treg function can lead to overactivation of IL-17-producing γδ T cells and subsequent uncontrolled inflammation (19). Our study provides the first systematic evidence of a concomitant reduction of γδ T cells and Treg in the peripheral blood of children with RMPP. This co-deficiency suggests a profound imbalance within the immunoregulatory network: deficiency in γδ T cells may compromise early anti-*M. pneumoniae* immunity, whereas diminished Treg may exacerbate inflammatory tissue damage. Owing to their unique functions, γδ T cells are emerging as potential immunotherapy targets; for example, chimeric antigen receptor-engineered γδ T cells can enhance targeted cytotoxicity. However, clinical translation of such approaches is currently hampered by challenges including *ex vivo* expansion difficulties, insufficient persistence, and potential off-target toxicity (23).

### 4.5 Integration of immunophenotypic findings with existing RMPP literature

Rather than focusing on conventional markers of inflammatory or tissue damage markers (e.g., C-reactive protein, neutrophil-to-lymphocyte ratio, lactate dehydrogenase, and D-dimer) (24–26), our study directly delineates the underlying immune dysregulation in RMPP at the level of immune cell functional phenotypes. Although we did not specifically aim to these traditional markers, differences in study design, sample characteristics, detection methods, mono-infection, and the inherent complexity of RMPP may account for this divergence in emphasis. Nevertheless, the immune characteristics we identified—such as the increased proportion of exhausted T cells (CD4 TEMRA and CD8 TEMRA) and reduced counts of B cell subsets—may partially explain the pathophysiological basis underlying these nonspecific indicators.

Our findings align with and extend the predictive logic of existing RMPP clinical prediction models, such as nomograms (27–29), by providing mechanistic corroboration at the immune phenotype level. The detailed immunophenotyping data from this study could thus serve as a key component for future multidimensional prediction models that integrate clinical, traditional laboratory, and immunological parameters. Li et al. reported that absolute counts of CD3+, CD4+, and CD19+ lymphocytes served as single-marker predictors for distinguishing RMPP from CMPP, with AUC values of 0.866, 0.900, and 0.842, respectively (11). Li et al. diagnosed MPP via serology (MP-IgM/IgG) and did not exclude co-infections with other bacteria or viruses. This approach may introduce confounding factors affecting immune profiles. In contrast, our study used nucleic acid testing with rigorous exclusion of mixed infections, ensuring more specific immunophenotypic characterization of RMPP. Our study presents a combined diagnostic model integrating four independent predictors—age, fever duration, pleural effusion, and absolute plasmablast count—achieving an AUC of 0.81 (95% CI: 0.74–0.88). Notably, although the individual AUC of plasmablast count (0.65) was modest, its inclusion alongside clinical parameters yielded a sensitivity of 96% at a specificity of 52%, offering a multidimensional framework that integrates both clinical and immunological dimensions of RMPP. Thus, unlike single-biomarker strategies, our model provides a more integrated approach for early risk stratification. It should be noted that the predictive model exhibited a relatively low specificity (52%), which limits its utility as a confirmatory tool; however, its high sensitivity (96%) suggests that it may serve as a sensitive screening tool to identify children at risk of developing RMPP in clinical practice. Nevertheless, because of its modest specificity, the model should not be used alone for clinical decision-making; rather, it could be combined with other clinical and laboratory parameters.

### 4.6 Clinical implications and future directions

This study provides a detailed characterization of the immune landscape in children with RMPP, defined by widespread reductions in T and B lymphocytes, an increased proportion of CD4 TEMRA and CD8 TEMRA, and a deficiency of immunoregulatory cells such as Treg and γδ T cells. This state of concurrent immunosuppression and exhaustion has several important implications for the clinical management of RMPP.

#### 4.6.1 Immune assessment for risk stratification

The distinct immune profile of RMPP suggests early, and detailed lymphocyte immunophenotyping could aid in identifying high-risk children, enabling more precise disease assessment and risk stratification.

#### 4.6.2 Optimizing immunomodulatory therapy

While glucocorticoids (e.g., pulse therapy) are important for controlling excessive inflammation in RMPP (30), our findings of concurrent immune exhaustion and suppression underscore the need to balance anti-inflammatory effects against the risk of exacerbating immunosuppression. Future efforts should therefore explore individualized strategies guided by immune phenotyping and investigate combination therapies involving immunomodulators (e.g., agents targeting T cell exhaustion) to optimize efficacy and safety.

#### 4.6.3 Expanding adjunctive therapeutic avenues

Beyond conventional antibiotics and corticosteroids, our findings support exploring adjuvant strategies aimed to correcting immune dysregulation. Potential approaches include reversing T cell exhaustion, enhancing B cell function, or employing agents such as intravenous immunoglobulin to restore effective anti-infective immunity.

### 4.7 Limitations and future perspectives

Despite offering valuable insights, this study has several limitations. Its single-center, retrospective design and relatively limited sample size warrant validation in larger, multicenter prospective cohorts. External validation of the predictive model in a separate, preferably multicenter, cohort is strongly recommended and currently being planned by our research group. A limitation of this retrospective study is that samples were collected at varying time points relative to symptom onset, although all were obtained at admission after refractoriness criteria were met. Because immunophenotypes evolve dynamically during infection, this heterogeneity may have influenced the observed absolute counts and subset distributions, as well as the performance of the predictive model. To establish whether the identified immune parameters (e.g., plasmablast count) can serve as true predictive biomarkers before refractoriness develops, prospective cohort studies with early standardized sampling – ideally within the first 48−72 hours of illness or at diagnosis of *M. pneumoniae* pneumonia – are urgently needed. The present model is hypothesis−generating and requires external validation in independent cohorts. Future research should also integrate detailed immune profiling analysis of the lower respiratory tract microbiome—considering its reported changes before and after COVID-19 pandemic (31)—host genetic factors, and the rising prevalence of macrolide-resistant *M. pneumoniae* with detailed immune profiling (32, 33). Applying multi-omics approaches (e.g., scRNA-seq, genomics, proteomics, metabolomics) will be essential for deeper investigation into the upstream drivers and signaling pathways underlying the observed immune phenotypes. Such efforts are crucial to establish a foundation for precise immune-based diagnosis and individualized therapy for RMPP.

## 5 Conclusion

In summary, this study systematically delineates a distinct landscape of adaptive immune dysregulation in children with RMPP, characterized by profound numerical depletion of T and B lymphocytes, an increased proportion of CD4 TEMRA and CD8 TEMRA, and a concomitant deficiency of immunoregulatory populations. This state represents not merely a consequence of excessive inflammation, but a complex condition of concurrent immunosuppression and functional exhaustion—an immune signature of “imbalance, exhaustion, and suppression” that constitutes a core component of RMPP immunopathology These findings provide a critical theoretical foundation and identify potential targets to advance early diagnosis, refine risk stratification, and guide the development of novel immunomodulatory adjunctive therapies for RMPP.

## Data Availability

The data that support these findings of this research are available from the corresponding author upon reasonable request.
