# Supplementary material for: Profound immune suppression and exhaustion characterize refractory mycoplasma pneumoniae pneumonia in children
Source: Front Immunol. 2026 Jun 9;17:1839837. doi: 10.3389/fimmu.2026.1839837 (PMC13286768; doi:10.3389/fimmu.2026.1839837)
Supplement: Supplementary file 1 [file Table1.docx]

**Supplementary Table S1** Comparison of percentages of lymphocyte subsets between children with refractory *Mycoplasma pneumoniae* pneumonia (RMPP) and those with common *Mycoplasma pneumoniae* pneumonia (CMPP)

| Cell subset (%) | RMPP (n=72) | CMPP (n=67) | p-value |
| --- | --- | --- | --- |
| Total T lymphocytes | 68.50 (63.00-73.00) | 69.00 (63.00-74.00) | 0.486 |
| CD4/CD8 ratio | 1.26 (1.04-1.91) | 1.52 (1.24-1.95) | 0.124 |
| CD4 | 34.92 ± 8.32 | 37.61 ± 7.63 | 0.049 |
| CD4 naïve | 69.30 (57.43-75.28) | 70.20 (64.60-78.50) | 0.069 |
| CD4 TEMRA | 0.55 (0.13-1.28) | 0.20 (0.10-0.50) | 0.008 |
| CD4 CM | 26.91 ± 10.09 | 24.53 ± 9.17 | 0.149 |
| CD4 EM | 4.05 (2.43-7.18) | 2.80 (1.50-5.40) | 0.007 |
| CD8 | 25.00 (21.00-32.00) | 24.00 (20.00-29.00) | 0.124 |
| CD8 naïve | 70.65 (54.03-79.58) | 75.20 (63.80-88.44) | 0.062 |
| CD8 TEMRA | 5.70 (1.00-17.93) | 2.00 (0.50-11.40) | 0.012 |
| CD8 CM | 17.04 ± 7.42 | 17.02 ± 7.60 | 0.986 |
| CD8 EM | 3.65 (0.90-7.73) | 2.40 (0.60-5.90) | 0.098 |
| DPT | 0.20 (0.10-0.40) | 0.20 (0.10-0.30) | 0.021 |
| DNT | 1.20 (0.90-1.50) | 1.10 (0.80-1.50) | 0.367 |
| Regulatory T | 9.20 (7.70-10.78) | 8.80 (7.60-9.90) | 0.678 |
| γδ T | 8.95 (6.15-13.23) | 8.30 (6.30-12.40) | 0.878 |
| Total B lymphocytes | 18.02 (12.32-25.74) | 18.56 (14.97-26.55) | 0.330 |
| Memory B | 13.95 (10.58-22.88) | 15.40 (10.20-20.50) | 0.878 |
| Naïve B | 76.25 (67.73-83.05) | 76.20 (67.10-81.00) | 0.773 |
| Transitional B | 5.20 (4.55-5.70) | 5.20 (4.10-5.80) | 0.934 |
| Plasmablasts | 0.75 (0.20-1.75) | 1.10 (0.30-3.40) | 0.100 |
| Natural killer | 12.0 (7.00-17.75) | 9.00 (7.00-13.00) | 0.065 |

Data are presented as median (IQR) or mean ± SD as indicated.

CM: central memory; DNT: double-negative T cells; DPT: double-positive T cells; EM: effector memory; ns: no significance; TEMRA: terminally differentiated effector memory T cells re-expressing CD45RA.
