# Supplementary material for: Profound immune suppression and exhaustion characterize refractory mycoplasma pneumoniae pneumonia in children
Source: Front Immunol. 2026 Jun 9;17:1839837. doi: 10.3389/fimmu.2026.1839837 (PMC13286768; doi:10.3389/fimmu.2026.1839837)
Supplement: Supplementary file 2 [file Table2.docx]

**Supplementary Table S2** Comparison of absolute counts of lymphocyte subsets between children with refractory *Mycoplasma pneumoniae* pneumonia (RMPP) and those with common *Mycoplasma pneumoniae* pneumonia (CMPP)

| Cell subset (cells/μL) | RMPP (n=72) | CMPP (n=67) | p-value |
| --- | --- | --- | --- |
| Total lymphocytes | 2251.09 (1682.10-3194.06) | 2812.84 (2115.29-3896.93) | 0.002 |
| Total T lymphocytes | 1656.14 ± 853.24 | 2137.99 ± 1038.07 | 0.003 |
| CD4 | 870.86 ± 474.84 | 1199.63 ± 630.84 | 0.001 |
| CD4 naïve | 506.52 (328.64-836.42) | 753.61 (497.35-1171.81) | 0.001 |
| CD4 TEMRA | 4.20 (1.10-9.63) | 2.74 (1.04-7.50) | 0.307 |
| CD4 CM | 217.61 ± 112.56 | 262.08 ± 118.46 | 0.025 |
| CD4 EM | 26.68 (18.74-50.83) | 26.18 (17.50-46.36) | 0.598 |
| CD8 | 578.19 (353.98-910.66) | 703.24 (518.31-925.94) | 0.057 |
| CD8 naïve | 344.80 (227.40-566.33) | 470.47 (327.49-739.00) | 0.007 |
| CD8 TEMRA | 32.21 (5.67-109.60) | 11.55 (3.96-80.25) | 0.082 |
| CD8 CM | 83.34 (57.44-129.84) | 109.47 (61.75-164.78) | 0.076 |
| CD8 EM | 17.06 (4.77-54.07) | 13.20 (5.83-39.31) | 0.483 |
| DPT | 2.92 (2.04-6.17) | 3.67 (1.76-5.41) | 0.928 |
| DNT | 16.13 (9.31-29.12) | 19.45 (12.85-34.70) | 0.108 |
| Regulatory T | 144.43 (89.86-196.60) | 181.00 (120.77-257.02) | 0.009 |
| γδ T | 134.46 (88.44-209.28) | 182.48 (108.39-253.61) | 0.022 |
| Total B lymphocytes | 379.31 (245.77-702.72) | 574.57 (328.26-838.45) | 0.007 |
| Memory B | 57.90 (36.03-89.90) | 80.68 (47.08-117.38) | 0.013 |
| Naïve B | 296.14 (170.24-536.97) | 444.20 (213.45-703.79) | 0.018 |
| Transitional B | 18.13 (11.62-32.49) | 28.93 (14.51-44.06) | 0.030 |
| Plasmablasts | 2.59 (1.06-5.88) | 7.13 (2.22-16.90) | 0.002 |
| Natural killer | 287.77 ± 191.41 | 317.93 ± 203.41 | 0.369 |

Data are presented as median (IQR) or mean ± SD as indicated.

CM: central memory; DNT: double-negative T cells; DPT: double-positive T cells; EM: effector memory; ns: no significance; TEMRA: terminally differentiated effector memory T cells re-expressing CD45RA.
